# Elaboration of Stable and Antibody Functionalized Positively Charged Colloids by Polyelectrolyte Complexation between Chitosan and Hyaluronic Acid

**DOI:** 10.3390/molecules18078563

**Published:** 2013-07-19

**Authors:** Ramona C. Polexe, Thierry Delair

**Affiliations:** 1 Cytosial Biomedic, 442 Rue Georges Besse, 30035 Nimes, France; E-Mail: rpolexe@cytosial.com; 2 Ingénierie des Matériaux Polymères, UMR CNRS 5223, Université Claude Bernard Lyon1, 15 Bd Latarjet, 69622 Villeurbanne, France

**Keywords:** chitosan, hyaluronic acid, polyelectrolyte complexes, antibody IgA

## Abstract

In this study, we describe the elaboration of multifunctional positively charged polyelectrolyte complex (PEC) nanoparticles, designed to be stable at physiological salt concentration and pH, for effective targeted delivery. These nanoparticles were obtained by charge neutralization between chitosan (CS) as polycation and hyaluronic acid (HA) as polyanion. We showed that the course of the complexation process and the physico-chemical properties of the resulting colloids were impacted by (i) internal parameters such as the Degree of Acetylation (DA, *i.e.*, the molar ration of acetyl glucosamine residues) and molar mass of CS, the HA molar mass and (ii) external parameters like the charge mixing ratio and the polymer concentrations. As a result, nonstoichiometric colloidal PECs were obtained in water or PBS (pH 7.4) and remained stable over one month. The polymer interactions were characterized by thermal analysis (DSC and TGA) and the morphology was studied by scanning electron microscopy. A model antibody, anti-ovalbumine (OVA) immunoglobulin A (IgA) was sorbed on the particle surface in water and PBS quantitatively in 4 h. The CS-HA/IgA nanoparticles average size was between 425–665 nm with a positive zeta potential. These results pointed out that CS-HA can be effective carriers for use in targeted drug delivery.

## 1. Introduction

Over the last years, polyelectrolyte complexes (PECs), prepared by electrostatic interactions between oppositely charged polyions, have received considerable attention, especially as carrier systems for drugs [1] or gene delivery [2]. The preparation of PEC nanoparticles is quite simple and can be easily performed under mild conditions without the use of either toxic organic solvents or chemical cross-linkers. Many factors influence the complex formation and the physical properties, such as the degree of ionization of polyions [3], pH, temperature, time of interaction and ionic strength [4]. The ability of a polycation-polyanion pair to form stable PECs may also be influenced by the local polymer chain flexibility, depending upon the relative molecular weights and the charge mixing ratio, as discussed by Schatz *et al.* for the chitosan-dextran sulfate system [5].

The aim of this work was to develop a targeted drug delivery system using a polyelectrolyte complex of chitosan and hyaluronic acid, two polysaccharides of the family of glycosaminoglycans. Chitosan (CS), is a polysaccharide obtained from partial deacetylation of chitin, hence it is a copolymer of N-acetylglucosamine and glucosamine and the molar fraction of remaining N-acetyl groups is called the Degree of Acetylation (DA) and has a strong impact on the physico-chemical properties of chitosan solutions [5]. Chitosan has some properties particularly attractive for medical applications such as: biocompatibility [6], biodegradability [7], nontoxicity [8] and it is rather inexpensive [9] as it is considered as a valorization product of biomass. The primary amine groups (–NH_2_) of the glucosamine residues can be protonated in weak acidic environments; therefore chitosan can form PEC nanoparticles with various polyanions such as dextran sulfate [10], chondroitin sulfates [11], alginate [12], carboxymethyl cellulose [13], carrageenan [14], polygalacturonic acid [15] and DNA [16]. 

Hyaluronic acid (HA) is a weak polyacid with a low charge density as only one charge is present for every two residues [17]. HA is a component of the extracellular matrix of all higher animals, has high capacities for lubrication, water sorption and water retention, which influences several cellular functions, such as migration, adhesion and proliferation [18]. Biomedical applications of hyaluronic acid include ophthalmic surgery, arthritis treatment, scaffolds for wound healing, tissue engineering and the use as a component in implant materials [19,20,21]. Hyaluronic acid can be used to form PECs with other polymers such as poly-L-lysine [22] and silk fibroin [23] for biomedical applications. 

Recently, chitosan/hyaluronic acid PECs nanoparticles were investigated as carriers for gene delivery [24,25]. CS-HA plasmid–DNA nanoparticles were synthesized through the complex coacervation of the cationic polymer with genes [24]. The average viability of cells transfected with CS-HA/plasmid nanoparticles was over 90%, suggesting that the nanoparticles could be an effective non-viral vector. Trimethylchitosan-hyaluronic acid PEC nanoparticles loaded with ovalbumin (OVA) were prepared by ionic gelation for nasal and intradermal vaccination [26]. These OVA-loaded nanoparticles had a size of around 250–350 nm, a positive zeta potential and OVA loading efficiencies up to 60%. Chua *et al.* [27] functionalized CS-HA polyelectrolyte multilayers (PEM) and immobilized RGD-containing peptide on PEM substrates to achieve enhanced osteoblast functions while retaining antibacterial efficacy.

Taking into account this information and the previous experience of our group [28] on PEC nanoparticles, the present study aimed at combining the virtues of CS and HA in the development of PEC nanoparticles for antibody immobilization. Hence, we focused on the elaboration and characterization of several non-stoichiometric polyelectrolyte complexes in order to obtain nanoparticles stable in physiological pH and salt concentration. To this end, the impacts on the course of the complexation process, of the internal parameters such as DA and molar mass of chitosan, hyaluronic acid molar mass and complexation conditions (molar mixing ratio, polymer concentration) were investigated by monitoring the sizes and polydispersities of the colloids and by measurement of zeta potentials. Furthermore, the sorption of anti-OVA IgAs was possible and afforded stable colloids with active recognition properties.

## 2. Results and Discussions

### 2.1. Physico-Chemical Properties of Chitosan and Hayluronic Acid

To study the formulation of polyelectrolyte complexes, chitosans of various degrees of acetylation and molar masses were synthesized. All physicochemical data related to the chitosan samples are reported in Table 1.

**Table 1:** Physicochemical characteristics of chitosan determined by SEC and NMR.

Chitosan								
Low Molar Masses			Medium Molar Masses			High Molar Masses		
M_w_ (g/mol)	DA (%)	PI	M_w_ (g/mol)	DA (%)	PI	M_w_ (g/mol)	DA (%)	PI
50 × 10^3^	5	1.56	100 × 10^3^	5	1.67	470 × 10^3^	5	1.72
30 × 10^3^	48	1.61	130 × 10^3^	48	1.47	430 × 10^3^	48	1.52
			250 × 10^3^	5	1.43			
			200 × 10^3^	48	1.54			

**Figure 1:**
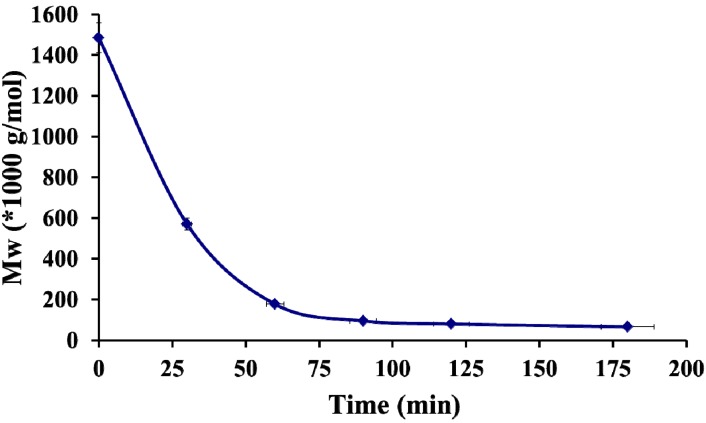
Kinetics of hyaluronic acid depolymerization, [HA] = 1 mg/mL Data are the average of three independent experiments ± standard deviation.

Hyaluronic acid was depolymerized to obtain a range of molar masses from a high initial Mw = 1 500 × 10^3^ g/mol. Ultrasound waves generated strong velocity gradients, close to the collapsing cavitation bubbles, that broke the macromolecules into smaller fragments in solutions [29]. The depolymerization proceeded rapidly in the early stage of the reaction, but it slowed down with prolonged sonication (Figure 1).

After 3 h a HA with molar mass of 50 × 10^3^ g/mol was obtained, corresponding to the lowest degree of polymerization that could be reached by our experimental set-up. For the PEC formulation three molar masses of HA obtained by sonication were used: 580 × 10^3^ g/mol (PI 1.76), 120 × 10^3^ g/mol (PI 1.61), 50 × 10^3^ g/mol (PI 1.27) and compared to the initial high molar mass 1 500 × 10^3^ g/mol.

### 2.2. Formation of Polyelectrolyte Complexes

**Figure 2:**
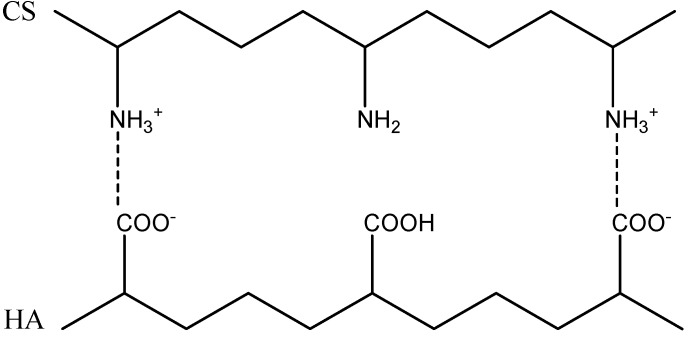
Schematic representation of the polyelectrolyte complexation reaction between chitosan (CS) and hyaluronic acid (HA).

The PECs were formed by the interaction between the ionized amino groups of chitosan (NH_3_^+^) and the ionized carboxyl acid groups (COO^−^) of hyaluronic acid (Figure 2). However, these electrostatic attractions can only occur when both polymers are ionized simultaneously. In all experiments, the initial solutions of chitosan were adjusted to pH = 4.0 to ensure full protonation [30]. But also, Denuzière *et al.* [31] observed that the full protonation of chitosan permitted to obtain PECs whatever was the pH of HA solution. 

Eight chitosan samples of low, medium, high molar masses and DAs of 5% and 48% were complexed with hyaluronic acid with average molar masses between 50 × 10^3^ g/mol and 1,500 × 10^3^ g/mol. The PECs were prepared a charge ratio R (n+/n^−^) = 1.5. The various colloidal dispersions obtained in this work were characterized by measuring their average sizes and size distributions after the complexation process. We carried out a detailed investigation of the influence of component characteristics.

**Figure 3:**
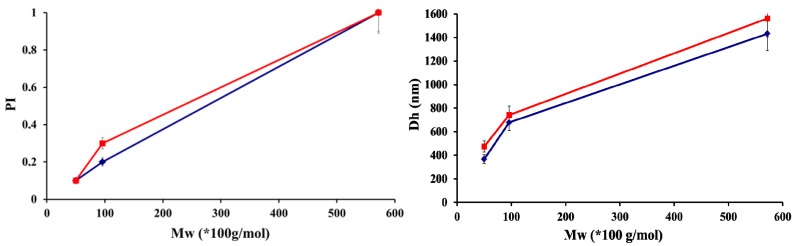
Influence of hyaluronic acid molar masses on sizes and polydispersities (PI) of polyelectrolyte complex particles for chitosan with DA 5% Mw = 100 × 10^3^ g/mol (■) and DA 48% Mw = 130 × 10^3^ g/mol (♦), at a charge ratio R = 1.5 and made in water. Datas are the average of three independent experiments ± standard deviation.

The increase in hyaluronic acid molar mass induced a significant increase in particle sizes (Figure 3). For HA sample of 1 500 × 10^3^ g/mol, no particles were formed; only precipitation occurred for all experiments. This suggested that such a high molecular weight polymer could not disentangle sufficiently enough to lead to individual particles, hence the aggregate formation. This was confirmed by the evolution trend depicted in Figure 3, which showed a concomitant increase in particle diameter and PI with hyaluronate molar mass. Therefore, the optimal sodium hyaluronate molar mass, for which particles displayed the lowest diameter and polydispersity index, was 50 × 10^3^ g/mol. In a recent paper, Umerska *et al.* [32] showed that the optimum molar mass for hyaluronic acid was 257 × 10^3^ g/mol, which is significantly higher than the optimal value of 50 × 10^3^ g/mol we obtained. Chitosan samples had similar molar masses for both investigations, though they are more isodisperse in our case.

**Figure 4:**
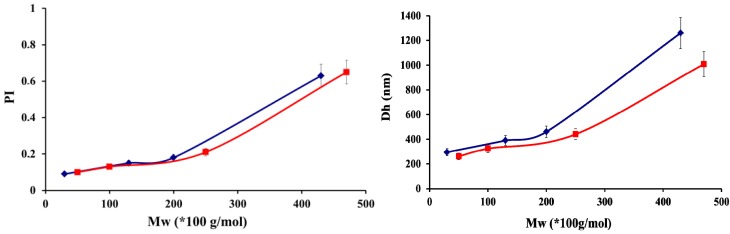
Influence of chitosan molar masses (Mw) on the sizes and polydispersities (PI) of polyelectrolyte complex particles for DA 5% (■) and 48% (♦) obtained with hyaluronic acid molar mass of 50 000 g/mol at a ratio R = 1.5 in water. Data are the average of three independent experiments ± standard deviation.

As displayed in Figure 4, an increase in the molar mass of chitosan increased the particle mean diameter for both DAs. Polydispersity indexes (PI) followed the same trend, providing evidence of the possible formation of larger structures with increasing chitosan molar masses, as recently suggested by Umerska *et al.* [32]. Our results show that a critical molar mass existed above which the increase in diameter was drastic: 250 × 10^3^ g/mol and 200 × 10^3^ g/mol for respectively DAs 5% and 48%. For molar masses of chitosan lower than the critical ones, the particles had a compact and uniform structure, as a result of a higher mobility of these polymer chains to achieve the conformational adaptation necessary to improve the charge matching.

To summarize, the formation of small and fairly isodisperse particles required low and medium molar mass chitosan (around 200 × 10^3^ g/mol and lower) and hyaluronic acid of molar masses below 50 × 10^3^ g/mol. Interestingly the DA of chitosan, *i.e.*, its charge density, had a limited impact on the course of the particle formation process.

As already stated [5], the formation of polyelectrolyte complexes on mixing solutions of polyanions and polycations is spontaneous and can lead to the formation of water-soluble complexes or precipitates, corresponding to two extreme cases. Aiming at obtaining PECs in the colloidal domain, non aggregating with time, we focused on the charge stoichiometry. Four charge ratios R (n^+^/n^−^): 0.5, 1.5, 2.5 and 3.5 were investigated for PEC nanoparticles obtained in water and PBS, for chitosan with a DA 48% and a molar mass of 130 × 10^3^ g/mol and HA whit low molar mass 50 × 10^3^ g/mol. The size, polydispersity index, zeta potential and appearance of these dispersions are reported in Table 2.

**Table 2:** Physicochemical characteristics of colloidal PECs after synthesis at different charge ratios. Chitosan (DA 48%, Mw 130 × 10^3^ g/mol) and hyaluronic acid (Mw 50 × 10^3^ g/mol).

CS-HA	Ratio (n^+^/n^−^)	Solid Content (%)	Size(nm)	PI	Zeta potential (mV)	Appearance
	0.5		1100	1		Precipitation
Water	1.5	6	350	0.1	30 ± 0.31	Medium turbidity
	2.5	3	290	0.1	35 ± 0.27	Medium turbidity
	3.5	1	271	0.1	47 ± 0.13	Low turbidity
	0.5		1220	1		Precipitation
	1.5	5	632	0.1	31 ± 0.19	Medium turbidity
PBS	2.5	3	424	0.2	48 ± 0.23	Medium turbidity
	3.5	1	418	0.1	49 ± 1.83	Low turbidity

For R (n^+^/n^−^) = 0.5 we observed a macroscopic flocculation, followed by a re-dissolution after 24 h. We can explain the observed re-dissolution by a rearrangement of the primarily formed complexes via a redistribution of the neutralized fragments involving more HA chains to allow the formation of the soluble complexes. So, this ratio was not retained for further investigations.

As already observed for chitosan/dextran sulfate colloidal PECs [5], the particle mean size increased significantly as the charge ratio approached 1, because of the complete charge neutralization. In the resulting particles, no electrostatic charge remained to counter-balance the particle association induced by Van der Waals interactions. As the charge ratio increased from 1, the particle size decreased for both buffers, as a result of an improved colloidal stability resulting from the increase in the magnitude of the net charge on the particles [33]. PEC nanoparticles with ratios R (n^+^/n^−^) from 1.5 to 3.5 had a diameter distribution was between 271–350 nm in water and 418–632 nm in PBS. The zeta potentials were positive and increased, as expected, with the charge ratio R by incorporation of an increasing number of chitosan chains in the colloidal macromolecular assembly. Therefore, the structure of the particle can be considered as core-shell with a relatively dense hydrophobic core, in which the charges are neutralized, surrounded by a much less dense hydrophilic shell containing the excess charges. Also, we can observe in Table 2 that the solid contents qualitatively decreased with increasing R, which can be attributed to the formation of water-soluble complexes in the presence of large excess of chitosan. These soluble complexes were removed from the colloidal dispersion during the centrifugation step following the particle formation step.

**Figure 5:**
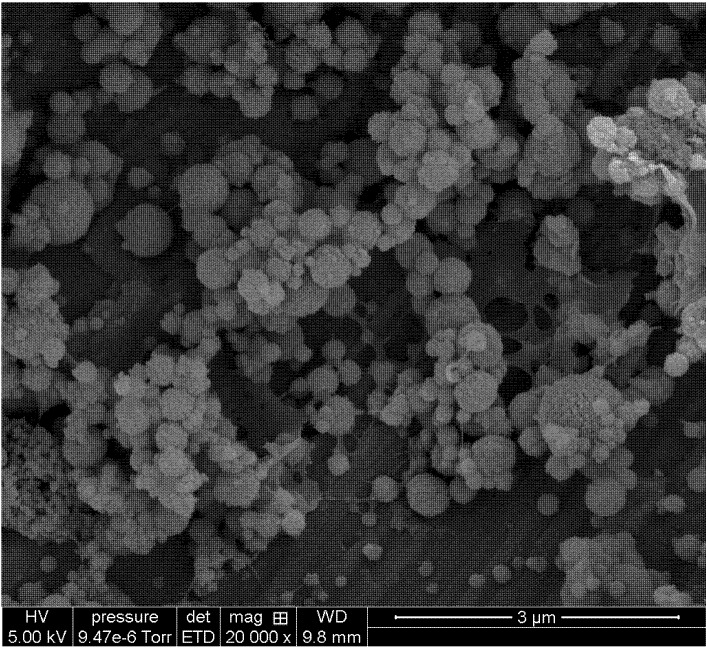
SEM CS-HA 0.01% in water (chitosan: DA 48%, molar mass 130 000 g/mol; hyaluronic acid 50 000 g/mol, R = 1.5).

The spherical morphology of chitosan-hyaluronic acid nanoparticles was established by SEM, Figure 5. The mean size of the CS-HA corresponded well to the values obtained by DLS. At R = 1.5, the particles displayed a noticeable rough surface.

**Figure 6:**
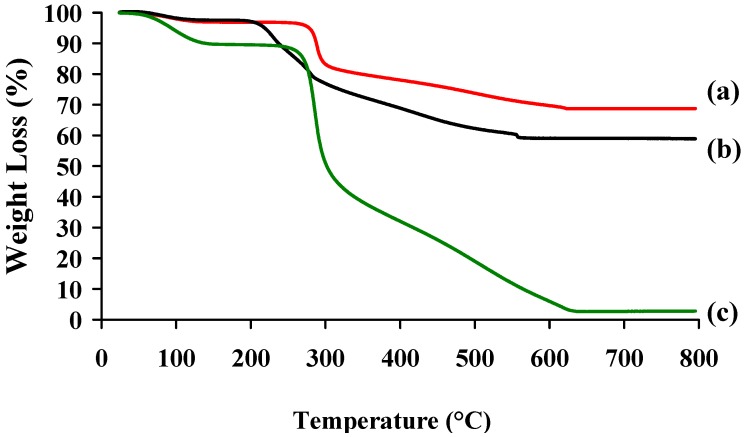
TGA profiles (**a**) CS-HA PEC complex, (**b**) HA and (**c**) CS.

The formation of a complex between chitosan and hyaluronic acid with a charge ratio R of 1.5 was checked by thermogravimetric analysis (TGA and DSC) in comparison with the two parent polymers. The TGA curves of CS-HA PECs and precursors (CS and HA) are shown in Figure 6. Two stages of mass loss were observed in the TGA curves of HA, CS, and CS-HA. The first one, ranging from 25 to 200 °C, was attributed to the elimination of water either weakly bound to the surface or trapped within the polymer chains. It is important to note that the samples showed different water contents between 4 to 10%. CS presented the highest water content (10%), HA and CS-HA showed the similar values (4–5%). In the second stage of weight loss, degradation occurred between 200 °C and 600 °C. The exact value of the sample degradation temperature was obtained through derivatives (DTG) of respective TGA curves. The degradation temperature of hyaluronic acid was estimated at 241°C and for chitosan at 298 °C. The PEC nanoparticles degradation temperature was about at 281°C, between that of HA and CS, confirming that the polymers were effectively associated and not simply physically blended. As a result, the CS-HA PECs nanoparticles were more thermally stable than HA, but less than CS [34], which is consistent with others works using chondroitine sulfate [11], peptide [35] or alginates [36] as polyanions.

**Figure 7:**
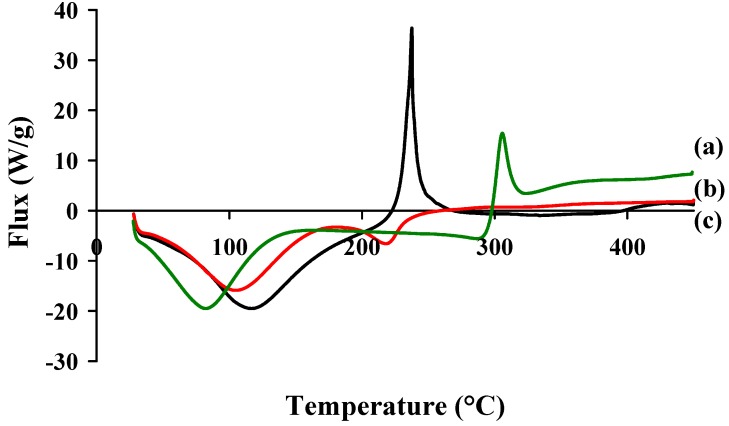
DSC profiles of (**a**) CS, (**b**) CS-HA PEC complex and (**c**) HA.

DSC curves are illustrated in Figure 7; all samples exhibited an endothermic peak between 90 °C and 150 °C attributed to the loss of water and/or a possible chain relaxation [37]. Exothermic peaks at 307 °C and 243 °C respectively showed CS and HA degradation. The thermal behaviour of CS-HA differed mainly in the intensity of the released heat during decomposition and also in the intensity of the peak maxima. There was a sharp endothermic peak at 230 °C corresponding to the occurrence of phase-transition and no clear exothermic peak was observed in the temperature range set in our experiment. As a result, the modification of HA with CS caused a shift of the endothermic peak and the particles had a lower decomposition temperatures, in comparison with HA. Moreover, it is suggested that more energy was needed for the decomposition of the nanoparticles compared with HA. As shown by these TGA and DSC results, the stability of the polyelectrolyte complex was lower than chitosan because the formation of strong electrostatic interactions between CS and HA charged groups induced the loss of the crystalline structure of chitosan, as shown by Denuzière [31].

### 2.3. Colloidal Stability

The colloidal stability was investigated by storing the particle dispersions at different solid contents for one month in water and PBS pH 7.4. The average particle diameters were measured by quasi-elastic light scattering. Most samples stored in aqueous solution were stable for at least 1 month though a noticeable increase in PI was observed at day 30 for R = 1.5 (Table 3).

**Table 3:** CS-HA nanoparticle colloidal stability with time as a function of buffers and charge ratio (chitosan: DA 48%, molar mass 130 × 10^3^ g/mol; hyaluronic acid 50 × 10^3^ g/mol).

CS-HA In water	Ratio (n^+^/n^−^) = 1.5		Ratio (n^+^/n^−^) = 2.5		Ratio (n^+^/n^−^) = 3.5	
Time (days)	Size (nm)	PI	Size (nm)	PI	Size (nm)	PI
0	350	0.1	291	0.1	270	0.1
7	332	0.1	256	0.1	265	0.1
14	280	0.2	233	0.1	204	0.1
30	181	0.3	204	0.2	112	0.2
**CS-HA In PBS**	**Ratio** **(n^+^/n^−^) = 1.5**		**Ratio** **(n^+^/n^−^) = 2.5**		**Ratio** **(n^+^/n^−^) = 3.5**	
Time (days)	Size (nm)	PI	Size (nm)	PI	Size (nm)	PI
0	632	0.1	424	0.2	418	0.1
7	621	0.1	410	0.2	368	0.1
14	588	0.1	376	0.2	340	0.1
30	332	0.3	243	0.2	254	0.2

The diameters measured in water were lower than in PBS buffer, reflecting the electrosteric mode of stabilization of the colloidal PECs. Upon protonation, the chitosan chains at the particle interface expanded into the aqueous external phase, preventing particles from aggregation. Screening the electrostatic charges, via the salts present in PBS for instance, the stabilization process was les efficient than in water, thus the particles had a higher mean diameter. Moreover, from the variations in diameters reported in Table 3, one can notice two important features. First, it was confirmed that on increasing the charge ratio R, the particle size was reduced, as the result of an improved stabilization mechanism during the particle formation step and storage. Second, we observed a decrease in particle diameter over time, irrespective of the conservation media and the values of the R ratio. This decrease can be attributed to a slow reorganization of the particles and /or a partial de-aggregation of aggregates formed during the centrifugation process. Anyhow, after one month of storing at room temperature, the polydispersity indexes had increased, as a proof of the broadening of the size distribution.

### 2.4. IgA Sorption

The sorption kinetics of anti-OVA IgAs were similar in both media and so were the maximum particle loading capacities, 62 and 54 mg/g (IgA/particles) for PBS and water respectively (Table 4). 

**Table 4:** IgA sorption yield, from protein solutions in water or PBS at different concentrations. CS-HA particles at a ratio R = 1.5 (CS Mw 130 × 10^3^ g/mol and DA = 48% and HA Mw = 50 × 10^3^ g/mol).

Time(h)	IgA input in PBS (µg/mL)			IgA input in water (µg/mL)		
	12	36	54	8	31	62
2	97	93	91	95	93	89
4	99	99	96	99	95	90
6	100	99	98	100	99	95
16	100	100	100	100	100	100
24	100	100	100	100	100	100

In the protein concentration range of this investigation, we did not reach the saturation of the area developed by the colloid. In other words, the higher the initial protein concentration, the more IgA was bound on the particle surface, with a quantitative yield obtained after 6 h of incubation. Altogether, these results emphasize the high affinity of the IgA for the colloids for both conditions. After antibody sorption onto the PECs, removal of the unbound proteins by centrifugation, and redispersion of the pellet, a second centrifugation process induced desorption of only around 1% of the total amount of proteins bound. Hence, IgAs were strongly bound to the colloidal structure.

The particle size after IgA sorption increased to 400–665 nm and remained stable in buffers for 3 weeks at room temperature, for particles with a solid content of 0.1%, kept under gentle stirring. The zeta potential of CS-HA nanoparticles after the incorporation of IgA varied from +24 mV to +27 mV in water and from +14 mV to +20 mV in PBS. The increased particle size and reduced zeta potential suggested that the antibodies were partially integrated into the nanoparticle structure, forming a semi-interpenetrating network via ionic interaction.

## 3. Experimental

### 3.1. Materials

Chitosan was obtained from squid pens chitin (Mahtani chitosan PVT, batch 113), with a Degree of Acetylation (DA), DA = 5%, Mw = 430 × 10^3^ g/mol. The sample was purified as follows: dissolution in acetic acid aqueous solution, filtration through Millipore membranes of decreasing porosity (from 3 to 0.45 nm), precipitation with ammonia, rinsing with deionized water until neutrality and freeze-drying. Purified high molar mass chitosans were N-acetylated in homogeneous medium with acetic anhydride. The reaction was performed in a hydro-alcoholic mixture according to the procedure previously described by Vachoud [38]. After re-acetylation, chitosan solutions were neutralized, rinsed with deionized water, and then freeze-dried. In addition, controlled nitrous deamination, allowing chain scissions [39] were carried out to produce low molar mass polymers. Chitosans (CS) were dissolved at 0.5% (w/v) in a 0.2 M acetic acid/0.1 M sodium acetate buffer. A 0.15 M sodium nitrite solution was added to chitosan solutions to obtain a nitrite/glucosamine unit molar ratio of 0.5. The reaction was performed under moderate magnetic stirring for various reaction times (15–60 min), depending on the targeted Mw. Chitosan was recovered by precipitation with ammonia and purified by several washings with deionized water until neutrality and finally freeze-dried. 

Hyaluronic acid sodium salt (HA) with high weight-average molar mass Mw =1,500 × 10^3^ g/mol was provided by H.T.L (Javené, France). Low molar mass HAs were obtained by sonication. The sonication was performed with an ultrasound Sonics Vibra Cell generator (Fisher Scientific Bioblock, Illkirch, France). The depolymerization was carried out in a glass reactor of diameter Φ = 3.5 cm and a maximum liquid height Hmax = 7 cm. Solutions were homogenized by magnetic stirring, and the reactor temperature was kept constant at 25 °C during experiments thanks to a water circuit. This method developed by Popa-Nita [40] *et al.* allowed us to obtain HA with different Mw: 580 × 10^3^ g/mol (PI 1.76), 120 × 10^3^ g/mol (PI 1.61), 50 × 10^3^ g/mol (PI 1.27). The immunoglobulin A (IgA) anti-OVA antibodies were provided by B-Cell Design (Limoges, France). Their concentration was confirmed by BCA Assay, according to the procedure provided by Pierce (Thermo Scientific, Hudson, NH, USA).

### 3.2. Chitosan Characterization

Degrees of acetylation were determined on purified chitosans by ^1^H-NMR spectroscopy (Varian, 500 MHz), according to the method developed by Hirai [41]. The weight-average molecular weight (Mw) and the polydispersity index (PI) were measured by size exclusion chromatography (SEC) (3.000 and 6.000 PW TSK gel columns, inner diameter = 7.8 mm and length = 300 mm) coupled on line with a differential refractometer (Waters 410) and a multi-angle-laser-light-scattering (MALLS, Wyatt) spectrophotometer equipped with a 5 mW He/Ne laser operating at k = 632.8 nm. Analyses were performed in micro-batch mode using the K5 flow cell. A degassed 0.2M acetic acid/0.15M ammonium acetate buffer (pH 4.5) was used as eluent. The flow rate was maintained at 0.5 mL/min. Refractive index increments (dn/dc) were determined from a master curve previously established under identical conditions, in the same solvent and with an interferometer (NFT ScanRef) operating at k = 632.8 nm.

### 3.3. Preparation of Polyelectrolyte Solutions

Chitosan was dispersed in Versol^®^ water or PBS medium at 0.1 wt % concentrations, taking into account the residual water. Dissolution was achieved under moderate stirring by adding a stoichiometric amount of acetic acid, with respect to the free amino functions. Then, solutions were adjusted to pH = 4.0 with 0.1M sodium hydroxide or hydrochloric acid. Before use, all solutions of chitosan were filtered on 0.22 µm pore size Millipore membranes. Hyaluronic acid solutions, at 0.1% concentration, were prepared directly in Versol^®^ water or PBS medium under magnetic stirring. 

### 3.4. Polyelectrolyte Complex Formation

Colloidal PECs were formed under non-stoichiometric conditions (R = n+/n^−^ 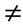
1, where *R* is the mixing molar charge ratio) by a one-shot addition of the polymer in default to the polymer in excess under magnetic stirring (750 rpm) at room temperature. The final particle dispersion volume was 30 mL at a solid content of 0.1 wt %. Under these conditions, the volume of excess polymer solution was always higher than the volume of the default polymer. Particles were separated from the polyelectrolyte mixture by centrifugation at 7 000× *g* and 20 °C for 10 min. The supernatant was removed and the pellet was re-suspended in deionized water or PBS.

### 3.5. Particle Solid Contents

The solid content was defined by the ratio between the weights of dried particles at 60 °C for 24 h, to the initial weight of the solution.

### 3.6. Physicochemical Characterization of the Complex Dispersions

Dynamic light scattering measurements of polyelectrolyte complex dispersions were carried out using a Malvern Nanosizer SZ equipped with a 10 mW He/Ne laser beam operating at *λ* = 633 nm (at 173° scattering angle). All measurements were performed at least in triplicate at 25 °C. The self-correlation function was expanded in a power series (Cumulants method). The polydispersity value provided by the software is a dimensionless value defined by *μ*_2_/(Γ)^2^, where *μ*_2_ is the second cumulant of the correlation function and (Γ) the average decay rate. Each measurement is the average of three series of 6 measurements each. For a monodisperse colloid, the polydispersity index should be below 0.05, but values up to 0.5 can be used for comparison purposes [42].

Zeta potentials were derived from electrophoretic mobility measurements using Smoluchowski’s equation. Electrophoretic mobilities (μE) of the particles were determined at 25 °C with the Malvern Nanosizer SZ. μE was expressed as the average of 10 measurements with a relative error of 5%. Electrophoretic mobilities were performed by suspending washed dispersions in 10^−3^ M sodium chloride solutions.

Thermal analyses DSC runs of PECs were performed on a calorimeter (Netzsch, Burlington, MA, USA). Samples of 2–10 mg were sealed in aluminium pans and heated from 20 to 450 °C at a rate of 10 °C min^−1^, under constant purging argon flow rate of 25 mL min**^−1^**.

TG-DTG analyses were carried out using a Setaram TGA (Caluire, France). 10–20 mg of samples were put in a ceramic pot and heated at a typical heating rate of 10 °C min^−1^ from room temperature up to 800 °C in a air atmosphere (flow rate: 20 mL min^−1^). 

Scanning Electron Microscopy (SEM) images were obtained using a Hitachi S-4800 microscope at 5 kV. A droplet of 0.01% (v/v) nanoparticles dispersion was deposited on a sample holder, air-dried at room temperature (12 h), and coated with palladium in a cathode evaporator (Technics Hummer II, Houston, TX, USA) under an argon atmosphere. The SEM was carried out at the ‘Centre Technologique des Microstructures’, Université Claude Bernard Lyon 1.

### 3.7. Antibody Sorption onto Colloidal PECs

The sorption process consisted in mixing equal volumes of particle dispersion CS-HA at a ratio R = 1.5 (CS with Mw 130 × 10^3^ g/mol and DA = 48%, HA Mw = 50 × 10^3^ g/mol) and antibody solution (IgA-antiOVA) under moderate end-overhead stirring. The antibody concentrations were obtained by dilution of the initial antibody solution with the same buffer. IgA/CS-HA particles were centrifuged 10 min at 14000g to remove potentially residual particles. The supernatant was separated; the pellet was resuspended in an identical buffer volume. Sorbed IgA was deduced from free IgA in the supernatant obtained by BCA assay titration, according to the manufacturer’s instructions, calibrated via serial dilutions in the same experimental buffer. The sorption yield was calculated as follows:

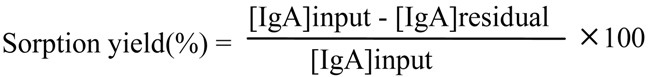



where [IgA] input is the IgA concentration titrated in the control sample (*i.e.*, all the reactants were present but not the particle suspension) of the original IgA solution for each independent experiment; [IgA] residual is obtained from titration of the supernatant, taking into account the background signal from a blank experiment in which all the reactants were present but the protein.

## 4. Conclusions

In this work positively charged polyelectrolyte complex (PEC) nanoparticles from hyaluronic acid and chitosan were obtained in such a way that they maintained their colloidal stability in physiological media for 30 days at room temperature. Moreover, antibodies could be sorbed quantitatively at the particle interface, with no alteration of the colloidal stability.

To achieve these very interesting nanoparticles, the following parameters were selected: low and medium molar mass chitosan (around 200 × 10^3^ g/mol and lower), hyaluronic acid of molar mass around 50 × 10^3^ g/mol and the charge ratio R (n^+^/n^−^) maintained in the 1.5 to 3.5 range. The process was carried out in deionized water or PBS and the particle solid content could be increased up to 5 w% after centrifugation and redispersion. The diameters of the obtained nanoparticles decreased with increasing R, but the conversion of the polymers into colloids concomitantly decreased. Interestingly the DA of chitosan, *i.e.*, its charge density, had a limited impact on the course of the particle formation process.

The association of anti-OVA IgA with chitosan-hyaluronic acid nanoparticles was characterized by fast kinetics and high loading capacities in water and PBS. After the IgA loading, the observed particle diameters were respectively 425nm and 665 nm in water and PBS, the zeta potentials remaining positive. CS-HA/IgA were stable for three weeks in both buffers. These results open the doors towards a great variety of applications in the delivery of bio-active (macro) molecules as particles were shown to feature a high loading capacity, to maintain their colloidal character on storing in physiological buffer (PBS) and were obtained out of non toxic polysaccharides from biomass. Further investigations are under way to elaborate a drug delivery nanosystem functionalized with antibodies for targeting.
